# Babesia, Theileria, Plasmodium and Hemoglobin

**DOI:** 10.3390/microorganisms10081651

**Published:** 2022-08-15

**Authors:** Daniel Sojka, Marie Jalovecká, Jan Perner

**Affiliations:** 1 Institute of Parasitology, Biology Centre, Academy of Sciences of the Czech Republic, Branišovská 1160/31, CZ-37005 České Budějovice, Czech Republic; 2 Faculty of Science, University of South Bohemia in České Budějovice, Branišovská 1645/31a, CZ-37005 České Budějovice, Czech Republic

**Keywords:** *Babesia*, *Theileria*, piroplasmida, *Plasmodium*, hemoglobin, malaria, babesiosis, piroplasmosis

## Abstract

The Propagation of *Plasmodium* spp. and *Babesia*/*Theileria* spp. vertebrate blood stages relies on the mediated acquisition of nutrients available within the host’s red blood cell (RBC). The cellular processes of uptake, trafficking and metabolic processing of host RBC proteins are thus crucial for the intraerythrocytic development of these parasites. In contrast to malarial *Plasmodia*, the molecular mechanisms of uptake and processing of the major RBC cytoplasmic protein hemoglobin remain widely unexplored in intraerythrocytic *Babesia/Theileria* species. In the paper, we thus provide an updated comparison of the intraerythrocytic stage feeding mechanisms of these two distantly related groups of parasitic Apicomplexa. As the associated metabolic pathways including proteolytic degradation and networks facilitating heme homeostasis represent attractive targets for diverse antimalarials, and alterations in these pathways underpin several mechanisms of malaria drug resistance, our ambition is to highlight some fundamental differences resulting in different implications for parasite management with the potential for novel interventions against *Babesia*/*Theileria* infections.

## 1. Introduction

Babesiosis (aka piroplasmosis) is a worldwide emerging malaria-like zoonosis. It is caused by parasites of the genus *Babesia,* belonging to the apicomplexan order Piroplasmida, a sister group to malaria parasites including Hemosporida [1]. Traditional Piroplasmida classification was based on morphological/life-history features, such as the presence (*Theileria* and *Cytauxzoon*) or absence (*Babesia*) of schizogony. However, a series of molecular phylogenetic studies over the past decade using nuclear and apicoplast sequences have shown that the genera of *Babesia* and *Theileria* are not monophyletic and that the genus *Cytauxzoon* is nested with the lineages that have been assigned to the former two genera [2]. In contrast to malarial *Plasmodia* that are transmitted by mosquitoes, *Babesia/Theileria* spp. are transmitted by ticks. The lifecycles of *Babesia/Theileria* and *Plasmodium* genera share some key features, such as asexual reproduction in vertebrate host erythrocytes and sexual reproduction in the midgut lumen of the arthropod vector serving as the definitive host [1]. Important differences in the intraerythrocytic cycles of *Babesia/Theileria* in comparison to malarial *Plasmodia* are the lack of synchrony of the intraerythrocytic life cycle, the absence of a parasitophorous vacuole, the absence of schizogony, and different mechanism of multiplication and egress [3].

Babesiosis is growing in incidence in both domesticated and wildlife animals [4]. Babesiosis is a severe and often fatal disease of livestock. Bovine babesiosis is the most economically important arthropod-transmitted disease of cattle, when up to 2 billion cattle worldwide are permanently exposed to potential infection, and outbreaks occasionally occur [5]. *Babesia divergens,* transmitted by the tick *Ixodes ricinus*, is the major causative agent of bovine babesiosis in Europe. Recently, increased attention has been devoted to the alarming increase in severe “dog babesiosis”, caused mainly by the larger *Babesia canis*, the smaller *Babesia gibsonii* and their related species transmitted by the ornate dog tick *Dermacentor reticulatus* [6].

Humans are accidental hosts of *Babesia*, but numerous factors contribute to human babesiosis as an emerging zoonosis. It shares many clinical features with malaria and can be fatal, particularly in the elderly and in immunocompromised individuals [7]. The majority of human infections are reported from the USA [8] where the principal agent of human babesiosis—*Babesia microti*—is one of the most common transfusion-transmitted pathogens [4]. In Europe, almost all reported cases of human babesiosis have been due to *B. divergens* infections that, in immunocompromised humans, can cause medical emergencies characterized by rapid fulmination and parasitemias that may exceed 70% [9]. Although the *Babesia* tick vectors and malaria vectoring mosquitoes live mostly in geographically separated areas, very little is known about the prevalence of *Babesia* spp. in overlapping regions due to the easy misdiagnosis of babesiosis as malaria [10,11]. Protection against bovine babesiosis has been based mostly on the vaccination of young cattle with attenuated parasites [12] and the treatment of babesiosis has relied on two common drugs—diminazen aceturate and imidocarb dipropionate. More recently, ivermectin has been demonstrated as an alternative remedy for animal *Babesia* infections [13]. The treatment of human babesiosis is non-specific and relies on antimalarial drugs and antibiotics such as Atovaquone and Azithromycin [8], with disease relapses and chronic infections occurring due to drug resistance in immunosuppressed patients. Alternative therapies with Clindamycin and Quinine are toxic and have never been tested in trials [14].

Due to this emergency and the lack of a *Babesia/Theileria*-specific treatment, novel intervention strategies against both animal and human piroplasmosis are highly desirable. One shortcut in the search for potential therapeutic targets is to look for *Babesia* analogues of malarial parasites. Hemoglobin serves as an essential source of nutrients for the intraerythrocytic development of malaria [15]. Cellular and molecular processes associated with its uptake and metabolism thus represent a target for diverse antimalarials and their activities underpin several mechanisms of drug resistance [16]. This review provides a short summary of major findings in the uptake and processing of host hemoglobin by the intraerythrocytic stages of malarial *Plasmodia* and aims to compare them with the relatively unexplored knowledge on utilizing host RBC proteins by the intracellular stages of *Babesia*/*Theileria*.

## 2. Hemoglobin Uptake and Processing in *Plasmodia*

*Plasmodium falciparum* internalizes large amounts of host red blood cell (RBC) hemoglobin. Yet, from about 65% of digested erythrocyte hemoglobin, the parasite utilizes only a small proportion, about 16%, of derived amino acids [17]. Such extensive proteolytic digestion of host cell cytoplasmic contents helps to prevent the lysis of infected erythrocytes prior to the completion of the intraerythrocytic asexual reproduction cycle. Thus, hemoglobin digestion clearly represents an indispensable process, not only providing sufficient nutrients but effectively helping to meet the space demands of the growing parasite and to balance the intracellular colloid osmotic pressure. The liberation of large quantities of free heme, which needs to be detoxified by its polymerization to crystalline hemozoin, generates a byproduct of rapid hemoglobin processing [18,19,20].

Surprisingly, despite decades of ongoing research, current knowledge about the cellular route(s) of host hemoglobin entry into food vacuole(s) during the intraerythrocytic stages of the malarial parasite lifecycle remains speculative. The substantial contribution by Elliot et al. [21], who used serial thin-section electron microscopy and three-dimensional reconstruction, identified four distinct pathways contributing to hemoglobin uptake and metabolism by the malarial parasite: (i) “Big Gulp” of ring-stage parasites resulting in food vacuole (FV) formation, (ii) plasma membrane and the parasitophorous vacuole membrane invagination through the cytostome (aka a micropore in other Apicomplexa) resulting in double membrane-bound small hemoglobin-containing vacuoles, (iii) cytostomal tubes that are long double membrane-bound tubular invaginations through the cytostome into the parasite cytoplasm and (iv) phagotrophs—large, actin-independent hemoglobin-uptake vesicles seen only in larger trophozoites [21]. In the last decade, multiple other studies based on different microscopic techniques reported hemoglobin-containing structures and reached conflicting conclusions [22,23,24,25]. Lazarus et al. [23] examined the ultrastructure of the endocytic apparatus during the intraerythrocytic stages of the *P. falciparum* lifecycle and concluded that hemoglobin uptake occurs by a vesicle-independent process. These authors reported that cytostomal invaginations elongate to form tubes that oppose the FV but remain connected to the parasite surface and open to the RBC cytoplasm. They suggested that the tubules pinch off from the parasite surface and simultaneously undergo fusion with the FV to release their contents into the FV lumen [23]. An important finding was made by Grüring et al. [26], who clearly demonstrated that there was no closure of the central cavity, a process termed the ‘Big Gulp’, which was proposed to create the single FV of trophozoites [21], and that the central cavity persists into the mid-trophozoite stage as a structure independent of the FV or the multiple FVs in earlier stages. Although the central cavity (aka the spherical structure) appears internal to the parasite, it is directly connected to the erythrocyte cytosol. The central cavity develops in the ring stage and persists until the parasite matures into a schizont. Digestive vacuoles are detected in close proximity to the central cavity, and although the functional significance of that is not understood, it does indicate that these compartments are separate entities [26].

Some of the most up-to-date insights into hemoglobin endocytosis in the malarial parasite were reported by Spielmann et al. [27], who finally focused a bright light onto this phenomenon. These authors reported that the molecular mechanism of uptake and processing of erythrocyte cytoplasm-containing hemoglobin is most-likely initiated by host cell cytosol-filled invaginations (cytostomes and phagotrophs) at the parasite periphery. This would imply that the “Big Gulp” theory, as originally proposed by Elliot et al. [21], might not be true. Instead, the molecular mechanism may rely on cytostomes, cytostomal tubules and phagotrophs, which are probably different components of one complex endocytic and possibly also excretory mechanism. There are two possible ways in which the intracellular endosomes and digestive vesicles may arise from the peripheral cytostomes and possibly also phagotrophs: the ‘cytostome maturation model’ proposes that the cytostome itself undergoes a cycle of initiation, maturation, and pinching off when the detached cytostome is transported to the FV. The alternative ‘cytostome hub model’ suggests that the cytostome serves as a hub that persists as a source of smaller endosomal vesicles that pinch off from the cytostome and are delivered to the FV or are collected in larger endosomal structures [27].

In *P. falciparum*, the FV apparently forms de novo after each round of infection [28]. The biogenesis, morphology, size and number of FVs vary between individual developmental stages of the parasite [29]. The multiple small dense vacuoles (150–300 nm in diameter) in early and mid-stage rings are formed by micropinocytosis of the host cell stroma from the parasite surface, which results in double membrane vesicles that function as individual FVs [30]. The inner membrane, which develops from the parasitophorous vacuole membrane (PVM), rapidly disappears, allowing more efficient nutrient diffusion into the parasite cytoplasm.

Late ring-stage malarial plasmodia (trophozoites) possess a cytostome, a cytoskeletal ring for the uptake of erythrocyte cytoplasm, and small pigment-containing vesicles coalesce to form a single large FV [29]. Notably, the structure and formation of the FV varies among the *Plasmodium* species: *P. falciparum* possesses a single FV, while *Plasmodium knowlesi* contains several smaller acidic compartments [31]. Differences occur between the cytostomal systems of murine *Plasmodia,* comprising long tubular structures connected with a branching tubular network, and that of *P. falciparum* showing vesicles budding off from the cytostome. These are consistent with parasite phylogeny as *P. falciparum* is significantly more related to avian than to other mammalian *Plasmodium* species (reviewed in Wunderlich et al. [29]).

Although the actual knowledge on the molecular background of malarial endocytic mechanisms remains limited, some interesting findings regarding molecules involved in host cell cytoplasm endocytosis by intracellular malarial parasites have recently been reported. The first protein with a confirmed role in host cell cytosol uptake is the *P. falciparum* orthologue of the vacuolar sorting protein 45 (VPS45). The conditional inactivation of PfVPS45 leads to limited parasite proliferation and the phenotype is complemented and rescued by an episomally expressed copy of PfVPS45 [32]. The loss of PfVPS45 function has no effect on the ring and young trophozoite stages but leads to arrest in trophozoites and schizonts [33]. PfVPS45-inducible knockdown (iKD) led to the accumulation of vesicles containing endocytosed material, prevented the delivery of this material to the parasite’s FVs and ultimately resulted in death of the parasite. The presence of phosphatidylinositol 3-phosphate on some of the accumulated vesicles indicates their endosomal character—a step before cargo degradation by FV proteases, as demonstrated by the lack of formation of hemozoin in these vesicles. This indicates that VPS45 is needed for the transport of host cell components to the FV and that the accumulated vesicles represent transport intermediates and not FVs. The VPS45 of mammalian and yeast cells typically functions with the early endosomal regulatory protein Rab5, when the endocytosed cargoes are delivered sequentially to the Rab5 compartment, the Rab7 compartment and finally to the lysosome [34]. *P. falciparum* encodes three Rab5 family proteins [35]. Although the expression of the constitutively active mutant form of *Pf*Rab5a demonstrated the role of this molecule in hemoglobin uptake and transport [21], more recent work using conditional mislocalization or conditional knockout of Rab5a resulted in growth arrest of the schizont stage, but its role in trophozoites could not be confirmed [34]. Although the *Pf*VPS45 data hint at the involvement of classical endosomal proteins, it has been proposed that the endocytic mechanism of apicomplexan parasites—namely, *T. gondii*—might rather resemble that of plants, which initially deliver endocytosed cargoes to the trans-Golgi network (TGN), followed by sequential movement through the Rab5 compartment, the Rab7 compartment and finally the lysosome for degradation [36].

Important findings have also been made recently by teams studying *P. falciparum* strains resistant to Artemisinin (ART) and its derivatives, the recommended first-line drugs against malaria. It is known that hemoglobin degradation by-products activate ART, and decreased hemoglobin digestion reduces ART susceptibility [37]. The mode of action of ART resistance through reduced levels of endocytic uptake of host cell cytosol was demonstrated by Birnbaum et al. [38], who explained why ART resistance is primarily associated with point mutations in the parasite’s Kelch propeller protein Kelch13 [39]. They demonstrated a Kelch13 role in hemoglobin uptake in the early ring stages of *P. falciparum* via an unusual clathrin-independent endocytosis pathway involving the Kelch13-Eps15 compartment associated with the FV [38].

## 3. Hemoglobin Uptake during the Intraerythrocytic Stages of *Babesia*

The apicomplexan order Piroplasmida, represented mainly by *Theileria* and *Babesia* genera, is diverse, consisting of at least ten principal lineages, of which *Babesia* parasites constitute four clades: *Babesia sensu stricto* known as ‘true’ *Babesia* and three lineages—Percei, Western, and *B. microti*-like groups—collectively referred to as *Babesia sensu lato* [2]. Unlike *Plasmodia*, Piroplasmida do not produce hemozoin, the product of hemoglobin digestion. This has always been interpreted as they either digest hemoglobin in a more efficient way than malarial parasites, or that they do not utilize hemoglobin as a source of amino acids for their own proteosynthesis [40]. In contrast to *Plasmodia,* the intraerythrocytic stages of *Babesia* do not grow within a parasitophorous vacuole, which is rapidly lost after host cell invasion [41]. Moreover, *Theileria* parasites are characterized by schizogony in nucleated blood cells—monocytes and lymphocytes—prior red blood cell invasion, while *Babesia* parasites are believed to multiply exclusively in erythrocytes (reviewed in [42]). Accordingly, there is significant variation in the endocytic mechanisms of intraerythrocytic stages of these different groups of *Piroplasmida*. Many previous conventional fine structure studies concerning the feeding behavior of intraerythrocytic stages of these organisms have been carried out [40,41,42,43,44,45] and these include the detection of phagocytosis and pinocytosis, the formation of food vacuoles, the involvement of cytostomes and the detection of pigments resulting from the incomplete digestion of ingested host cell material. However, these studies have resulted in many diverse observations and interpretations.

**Figure 1:**
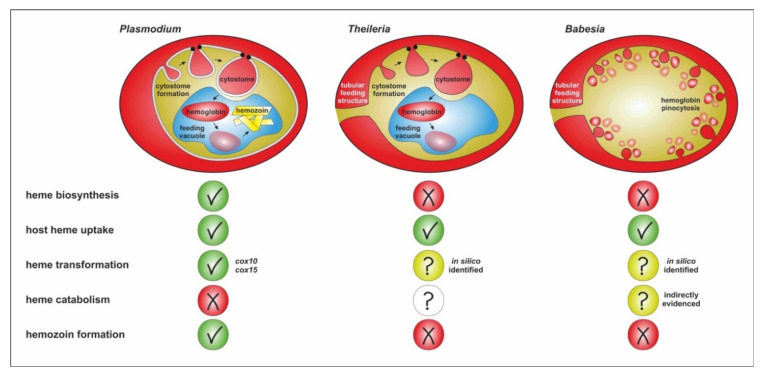
Schematic depiction of the major cellular differences of *Plasmodium*, *Theileria* and *Babesia* trophozoites uptaking and processing host RBC cytosol proteins, including the utilization and detoxification of resulting heme molecules. The heme transformation pathway has been functionally proven only in *Plasmodium*, but the direct homologues *cox*10 and *cox*15 genes (*cox* = cytochrome c oxidase) that encode the key enzymes of this pathway have been determined from the *Babesia/Theileria* genomes. Heme catabolism pathway is to date only indirectly evidenced in *Babesia* by the presence of a gene encoding a heme oxygenase-like homologue in the genome and the identification of higher carboxyhemoglobin fractions in dogs suffering from severe babesiosis [49].

Certain aspects of the feeding mechanism have been reported from the analysis of electron-micrographs of *Theileria equi* (formerly known as *Babesia equi*), including pinocytosis and the formation of FVs with similar content density as the host RBC cytoplasm. However, the cytostome, together with a tubular food vacuole, has been described in multiple *Theileria* species [44,45]. The tubular feeding structure is formed by the concomitant invagination of the erythrocyte plasma membrane (EM) by the parasite. Interestingly, this tubular structure extends from the interior of the trophozoite to the periphery of the host erythrocyte, where its lumen frequently maintains direct contact with host blood, thus interconnecting the parasite with the host extracellular space. Thus, it appears involved in direct feeding or excretion from/to host blood plasma [43]. Higuchi [44] observed similar tubules in *Theileria sergenti,* along with “excreta-like” material outside the RBC in the region where the tubule was in contact with the parasite. These authors argued that the parasite probably excretes actively from the tubular structure. Controversy persists, however, with regard to the role of this structure (ingestion or excretion), and further studies are required to determine its true function. We speculate that this tubular structure might be identical to the coiled membranous organelle composed of coiled multiple membranes and located partly outside and partly inside *B. microti* trophozoites, suggesting its involvement in feeding via the extracellular proteolysis of host cytoplasm [40,46]. This structure might be also identical with the later identified dendrite-like tubulovesicular structure (tubes of vesicles, TOVs) involved in the vesicular excretion of *Babesia duncani* [47], although it was originally described as a secretory organ for the intracellular remodeling of invaded host RBC not connected to the outer space (plasma). However, this contrasts with previous scanning EM observations displaying a single perforation on the surface of *B. microti* erythrocytes [48], confirming a connection of the parasite and the host plasma, most likely via the tubular feeding structure. In multiple piroplasmid species, this direct connection (Figure 1) seems to be part of the adaptation of specific endocytic and processing mechanisms of host cell proteins during intraerythrocytic survival. Although a similar situation has been initially described in several *Babesia* species, *Babesia* was later reported to not possess a cytostome and true FV [40].

The large dense bodies of the same structure and density as the host cytoplasm, initially considered as FVs, have been shown by serial sections to not contain internalized cytoplasm but only portions of host cytoplasm partially surrounded by the parasite; they were named pseudo-food vacuoles [40]. To our consideration, this classic work might pre-date the more recent findings in *Plasmodia* [26] demonstrating the presence of a FV-independent central cavity. This apparently represents a shared structural phenomenon among the piroplasm and plasmodia blood stages, where the FV-independent central cavity persists into the mid-trophozoite stage. These central cavities most likely serve to increase the surface area of the parasite–host cytoplasm interface, facilitating the ingestion of nutrients. Nevertheless, *B. microti* does not appear to feed by the phagocytosis of large boluses of hemoglobin, as does *Plasmodium*, but experiments performed with feeding this parasite with ferritin and albumin indicated that this *sensu lato Babesia* species pinocytoses hemoglobin in vivo [46]. This is in accordance with the morphological observations of intraerythrocytic *B. microti,* which apparently increases its surface area by multiple cell shape modifications, such as the appearance of numerous pseudopods, folds and invaginations. The presence of a single plasma membrane and enlarged surface contact with the erythrocyte might secure a faster and more efficient feeding by diffusion [40]. Indeed, both phagocytotic mechanisms involving cytostomes and tubular food vacuoles, as well as pinocytosis, are possible ways in which hemoglobin and other host RBC cytoplasmic proteins can be internalized by piroplasmid parasites. It is obvious that a more in-depth ultrastructural study of the cellular mechanisms of host cytoplasm uptake during intraerythrocytic development would be very useful. This study should ideally involve a variety of *Babesia* species. The results should be interpreted with respect to the actual phylogenetic position of each species [2] and put in context with the early fine structure studies of the *Babesia* feeding mechanism.

## 4. Hemoglobin Processing in *P. falciparum*

The processing of host hemoglobin occurs solely inside the acidic FV of *P. falciparum* intraerythrocytic stages [50] with an estimated pH of 5.0 to 5.4, maintained by H+ pumps [51]. It is separated into two distinct mechanisms—(i) the proteolytic degradation of the globin chains and (ii) utilization and detoxification of resulting heme groups (Figure 1). The first mechanism utilizes the protein part of adult hemoglobin A (HbA), consisting of a tetramer of two α-globins and two β-globins as the main protein source for the development and multiplication of the intraerythrocytic parasite. Proteolytic degradation takes place by the action of proteases operating at the optimal low pH of the FV [52]. Globin chain unwinding is initiated by aspartyl proteases (plasmepsins), followed by endopeptidolytic cleavage by papain-like cysteine proteases (falcipains, FP) [53,54]. *P. falciparum* possesses four digestive vacuole-residing hemoglobinolytic plasmepsins PfPMI–PfPMIV, and PfPMIII, which is more often referred to as the histo-aspartyl protease (HAP). However, all PfPM isoenzymes are close homologs. They form a cluster in a clearly distinguished single clade (A) of apicomplexan aspartyl proteases [55], together with digestive plasmepsins of other plasmodial species, represented by a single homologue in plasmodium species outside of the primate-infecting group, *P. falciparum* [56]. This parasite encodes four falcipain isoenzymes FP1, FP2a, FP2b, and FP3, out of which FP2a and FP3 appear to be the principal cysteine proteases involved in hemoglobin digestion in early trophozoites and later erythrocytic stages, respectively [57]. The best characterized are PfFP2a and PfFP2b, responsible for most of the cysteine protease activities in the FV [58]. Protein fragments liberated by digestive plasmepsins and falcipains are subsequently degraded to dipeptides and free amino acids by the exopeptidase activities of the metalloprotease falcilysin, and other aminopeptidases [50].

The digestion of one hemoglobin molecule releases four molecules of Fe^2+^ iron-containing reactive hem, which is oxidized into Fe^3+^. This is accompanied by an accumulation of reactive oxygen species (ROS) with the production of O_2_^−^, which still in the low pH FV is thought to dismutate spontaneously to H_2_O_2_ and O_2_ [59]. The mechanism of heme detoxification and potential utilization starts at the intersection of the concerted function of multiple digestive vacuole proteases and heme detoxification enzymes that assist in the conversion of hemoglobin-heme into hemozoin—the final form of heme metabolism in *P. falciparum* [60]. With hemoglobin processing, liberated heme is released into the acidic environment of the FV. The parasite detoxifies the free heme groups by its biomineralization, promoted by the FV low pH, into hemozoin [61]. In contrast to the knowledge of hemozoin composition, a considerable debate persists regarding hemozoin crystal formation that might involve membrane lipids [62], histidine-rich proteins [52], hem detoxification protein [63], and the transport protein lipocalin, involved in the motion and morphology of the hemozoin crystal [64,65]. However, not all acquired hemes are transformed into hemozoin, as a certain amount of host heme is recycled and re-used in heme-based metabolism of the parasite [66]. Despite coding for a complete heme biosynthetic pathway, encompassing eight enzymes, the biosynthetic pathway is dispensable in intraerythrocytic stages of the parasite, as knockouts of the last enzyme of the pathway ferrochelatase are viable at this parasitic stage [67,68]. Further studies, using inhibitors or gene disruptions, support the conclusion that parasites do not depend on heme biosynthesis during blood-stage infection [69]. Thus, it seems reasonable to speculate that *Plasmodium* opts to acquire exogenous heme when in a heme-rich environment, while it is capable of de novo heme synthesis when it passes through heme-scarce micro-environments during the complementation of its lifecycle [70].

## 5. Hemoglobin Processing in *Babesia*

The missing evidence of a true FV in *Babesia/Theileria* species and the lack of direct homologues of hemoglobin-digesting clade A plasmepsins has been used as an argument to postulate that *Babesia* is not able to utilize host RBC hemoglobin [71]. However, although *Babesia/Theileria* parasites do not encode direct homologues of *P. falciparum* FV plasmepsins [55], they do encode several papain-like cysteine proteases [54]. It has also been hypothesized that other aspartyl proteases of the A1 family are likely to be secreted into the erythrocyte cytoplasm and are able to initiate hemoglobin degradation there; such speculative statements would need further experimental exploration [72].

The identified *Babesia*/*Theileria* cysteine protease homologues of hemoglobinolytic *P. falciparum* falcipains are listed in Table 1. PfFP2 homologues are expressed in the intraerythrocytic stages of *Babesia bovis* and *Babesia bigemina* and are also released into the erythrocyte cytoplasm in a similar fashion to PfFP2 [73,74,75,76]. Recently, PfFP2 homologues were also found in *B. microti* [72]. However, the relatively far phylogenetic distance (maximum 35% identity to *P. falciparum* in primary protein structures) as well as the lack of biochemical and functional characterization defies the definition of the exact roles of these enzymes and their importance for host cell cytoplasmic protein processing during intraerythrocytic stages of *Babesia/Theileria*. To date, the only indirect evidence has been provided using cysteine protease inhibitors having a hampering effect on *B. bovis* erythrocyte invasion and in vitro replication [77], and from the reduced parasitemia observed in *B. ovis* erythrocyte cultures exposed to antibodies against ovipain-2 [78].

Of interest is the heme metabolism of *Babesia* and other piroplasms, as it apparently differs from malarial *Plasmodia*. Piroplasmid parasites do not encode heme biosynthetic enzymes [79,80], nor do they form hemozoin crystals [81]. This deviation from our intuitive anticipation of heme biology within parasites clearly deserves a detailed assessment. A clear impact is that, while the formation of hemozoin in malarial parasites is a valid drug target [61], with an array of very effective drugs and commercial antimalarials being available, *Babesia/Theileria* species are not sensitive to compounds such as chloroquine [82]. The absence of hemozoin pigments in later intraerythrocytic stages has two possible explanations: (i) hemoglobin is taken up in a tightly controlled mechanism so that no heme is left un-utilized after hemoglobin proteolysis, or (ii) hemoglobin uptake is copious in *Babesia*, similar to *Plasmodium*, yet the distinct biology of *Babesia,* with a clear localization and environment for hemoglobin proteolysis might lead to the formation of heme catabolic products, e.g., biliverdin, iron and carbon monoxide. The latter explanation is supported by (i) the identification of a heme oxygenase-like homologue in *Babesia microti* (GenBank XP_012649431.2), which may possibly form biliverdin [83], and (ii) the identification of higher carboxyhemoglobin fractions in dogs with severe babesiosis as a result of CO release upon heme cleavage. The superimposition of carboxyhemoglobinemia on severe anemia results in the further compromising of the oxygen status of dogs with severe babesiosis, and probably plays a role in the pathogenesis of hypoxic tissue damage associated with this condition [49]. The identification of ferritin within the coiled organelles of *Babesia* might indicate that it serves to mop up excess iron released from heme catabolism [46]. The heme biology of *Babesia/Theileria* thus warrants further investigation, as its unique features, together with clear implications for pathogenesis, may conceal novel drug targets in these parasites.

## 6. Summary and Outstanding Questions

Apicomplexan parasites share the unique capability of invasion, persisting and multiplication within other eukaryotic cells. Both related genera *Plasmodium* and *Babesia/Theileria* have adapted their strategies to multiplicate within host RBC representing an “all-you-can-eat buffet” for their haploid vertebrate stages. Although there are analogies in the initial phase of the intraerythrocytic multiplication cycles, there are significant differences in both the trafficking and processing of hemoglobin as the major protein component of the invaded cells. While *Plasmodium* spp. and at least some *Theileria* species possess the cytostome and food vacuole(s) as structures to acquire RBC cytosol containing hemoglobin, the uptake in *Babesia* spp. seems to be exclusively occurring through pinocytosis (Figure 1). Accordingly, the enzymatic machineries involved in the intracellular processing of host RBC proteins, including the globin chains of hemoglobin, seem to differ, as does the downstream management of free heme molecules released during hemoglobin digestion. Below, we define six outstanding questions resulting from this review that may help to pave the way for more in-depth knowledge of molecular adaptations, enabling the intraerythrocytic survival of piroplasmid parasites:

(1) Is the uptake and intracellular trafficking of hemoglobin by piroplasmid apicomplexans based on analogous molecular components/machineries as in *P. falciparum*?
(2) Does the appearance of the cytostome and the FV in *Theileria* and not in *Babesia* have anything to do with the ability to create multinucleate syncytium during intra-leucocytic schizogony prior to intraerythrocytic merogony?
(3) Is heme catabolized by intraerythrocytic *Babesia* stages, and if so, what products are formed?
(4) Does the absence of hemozoin formation prevent using anti-malarials, exploiting the formation of hemozoin to exert toxicity?
(5) Is there a link between the heme auxotrophy of ticks and the way of hemoglobin uptake and processing in *Babesia* and *Theileria* parasites, apparently differing from malarial plasmodia? Is this due to their co-evolution with the tick vector/definitive host?

## Figures and Tables

**Table 1:** Three molecules involved in the uptake and processing of hemoglobin in intraerythrocytic *P. falciparum* and their potential analogues in selected *Babesia*/*Theileria* species.

*P. falciparum* Protein	Description	Potential Analogue in Piroplasmida Species/GenBank Ref. No.
Kelch 13	Located at the cytostome surface, class of Kelch/BTB/POZubiquitination adaptors, hemoglobin uptake cytostomal invagination	*Babesia microti*/XM_012793132.1 *Babesia bovis*/XM_001608959.1 *Babesia bigemina*/XM_012912362.1 *Babesia ovata*/XM_029011617.1 *Theileria equi*/XM_004829658.1 *Theileria parva*/XM_760915.1 *Theileria oriantalis*/XM_009690968.1
VPS45	Vacuolar protein sorting-associated protein 45 role in the trafficking of ingested hemoglobin to FV for degradation	*Babesia microti*/XM_012794275.1 *Babesia bovis*/XM_001611295.1 *Babesia bigemina*/XM_012913205.1 *Babesia ovata*/XM_029009877.1 *Theileria equi*/XM_004832773.1 *Theileria parva*/XM_758502.1 *Theileria oriantalis*/XM_950220.1
falcipain-2(FP2)	Cysteine protease; degradation of globin chains within FV together with aspartyl proteases plasmepsins	*Babesia microti*/XM_012792174.1 *Babesia bovis*/GQ412131.1 *Babesia bigemina*/FJ859910.1 *Babesia ovata*/XM_029009877.1 *Theileria equi*/XM_004830311.1 *Theileria parva*/XM_758208.1 *Theileria oriantalis*/XM_009693310.1

## References

[1] Florin-Christensen M., Schnittger L. (2009). Piroplasmids and ticks: A long-lasting intimate relationship. Front. Biosci..

[2] Jalovecka M., Sojka D., Ascencio M., Schnittger L. (2019). *Babesia* life cycle–when phylogeny meets biology. Trends Parasitol..

[3] Sevilla E., González L.M., Luque D., Gray J., Montero E. (2018). Kinetics of the invasion and egress processes of *Babesia divergens*, observed by time-lapse video microscopy. Sci. Rep..

[4] Yabsley M.J., Shock B.C. (2013). Natural history of zoonotic *Babesia*: Role of wildlife reservoirs. Int. J. Parasitol. Parasites Wildl..

[5] Bock R., Jackson L., de Vos A., Jorgensen W. (2004). Babesiosis of cattle. Parasitology.

[6] Solano-Gallego L., Sainz Á., Roura X., Estrada-Peña A., Miró G. (2016). A review of canine babesiosis: The European perspective. Parasites Vectors.

[7] Djokic V., Primus S., Akoolo L., Chakraborti M., Parveen N. (2018). Age-related differential stimulation of immune response by *Babesia microti* and *Borrelia burgdorferi* during acute phase of infection affects disease severity. Front. Immunol..

[8] Vannier E., Krause P.J. (2012). Human babesiosis. N. Engl. J. Med..

[9] Zintl A., Mulcahy G., Skerrett H.E., Taylor S.M., Gray J.S. (2003). *Babesia divergens*, a bovine blood parasite of veterinary and zoonotic importance. Clin. Microbio. Rev..

[10] Gonzalez J., Echaide I., Pabón A., Gabriel Piñeros J.J., Blair S., Tobón-Castaño A. (2018). Babesiosis prevalence in malaria-endemic regions of Colombia. J. Vector Borne Dis..

[11] Bloch E.M., Kasubi M., Levin A., Mrango Z., Weaver J., Munoz B., West S.K. (2018). *Babesia microti* and Malaria Infection in Africa: A Pilot Serosurvey in Kilosa District, Tanzania. Am. J. Trop. Med. Hyg..

[12] Florin-Christensen M., Suarez C.E., Rodriguez A.E., Flores D.A., Schnittger L. (2014). Vaccines against bovine babesiosis: Where we are now and possible roads ahead. Parasitology.

[13] Batiha G.E., Beshbishy A.M., Tayebwa D.S., Adeyemi O.S., Yokoyama N., Igarashi I. (2019). Evaluation of the inhibitory effect of ivermectin on the growth of *Babesia* and *Theileria* parasites in vitro and in vivo. Trop. Med. Health.

[14] Renard I., Ben Mamoun C. (2021). Treatment of human babesiosis: Then and now. Pathogens.

[15] Counihan N.A., Modak J.K., de Koning-Ward T.F. (2021). How malaria parasites acquire nutrients from their host. Front. Cell Dev. Biol..

[16] Rocamora F., Winzeler E.A. (2020). Genomic approaches to drug resistance in malaria. Annu. Rev. Microbiol..

[17] Krugliak M., Zhang J., Ginsburg H. (2002). Intraerythrocytic *Plasmodium falciparum* utilizes only a fraction of the amino acids derived from the digestion of host cell cytosol for the biosynthesis of its proteins. Mol. Biochem. Parasitol..

[18] Esposito A., Choimet J.B., Skepper J.N., Mauritz J.M., Lew V.L., Kaminski C.F., Tiffert T. (2010). Quantitative imaging of human red blood cells infected with *Plasmodium falciparum*. Biophys. J..

[19] Hanssen E., Knoechel C., Dearnley M., Dixon M.W., Le Gros M., Larabell C., Tilley L. (2012). Soft X-ray microscopy analysis of cell volume and hemoglobin content in erythrocytes infected with asexual and sexual stages of *Plasmodium falciparum*. J. Struct. Biol..

[20] Goldberg D.E., Zimmerberg J. (2020). Hardly Vacuous: The parasitophorous vacuolar membrane of malaria parasites. Trends Parasitol..

[21] Elliott D.A., McIntosh M.T., Hosgood H.D., Chen S., Zhang G., Baevova P., Joiner K.A. (2008). Four distinct pathways of hemoglobin uptake in the malaria parasite *Plasmodium falciparum*. Proc. Natl. Acad. Sci USA.

[22] Bakar N.A., Klonis N., Hanssen E., Chan C., Tilley L. (2010). Digestive-vacuole genesis and endocytic processes in the early intraerythrocytic stages of *Plasmodium falciparum*. J. Cell Sci..

[23] Lazarus M.D., Schneider T.G., Taraschi T.F. (2008). A new model for hemoglobin ingestion and transport by the human malaria parasite *Plasmodium falciparum*. J. Cell Sci..

[24] Milani K.J., Schneider T.G., Taraschi T.F. (2015). Defining the morphology and mechanism of the hemoglobin transport pathway in *Plasmodium falciparum*-infected erythrocytes. Eukaryot. Cell.

[25] Wendt C., Rachid R., de Souza W., Miranda K. (2016). Electron tomography characterization of hemoglobin uptake in *Plasmodium chabaudi* reveals a stage-dependent mechanism for food vacuole morphogenesis. J. Struct. Biol..

[26] Grüring C., Heiber A., Kruse F., Ungefehr J., Gilberger T.W., Spielmann T. (2011). Development and host cell modifications of *Plasmodium falciparum* blood stages in four dimensions. Nat. Commun..

[27] Spielmann T., Gras S., Sabitzki R., Meissner M. (2020). Endocytosis in *Plasmodium* and *Toxoplasma* parasites. Trends Parasitol..

[28] Tonkin C.J., Pearce J.A., McFadden G.I., Cowman A.F. (2006). Protein targeting to destinations of the secretory pathway in the malaria parasite *Plasmodium falciparum*. Curr. Opin. Microbiol..

[29] Wunderlich J., Rohrbach P., Dalton J.P. (2012). The malaria digestive vacuole. Front. Biosci..

[30] Dluzewski A.R., Ling I.T., Hopkins J.M., Grainger M., Margos G., Mitchell G.H., Holder A.A., Bannister L.H. (2008). Formation of the food vacuole in *Plasmodium falciparum*: A potential role for the 19 kDa fragment of merozoite surface protein 1 (MSP1(19)). PLoS ONE.

[31] Fulton J.D., Flewett T.H. (1956). The relation of *Plasmodium berghei* and *Plasmodium knowlesi* to their respective red-cell hosts. Trans. R. Soc. Trop. Med. Hyg..

[32] Birnbaum J., Flemming S., Reichard N., Soares A.B., Mesén-Ramírez P., Jonscher E., Bergmann B., Spielmann T. (2017). A genetic system to study *Plasmodium falciparum* protein function. Nat. Methods.

[33] Jonscher E., Flemming S., Schmitt M., Sabitzki R., Reichard N., Birnbaum J., Bergmann B., Höhn K., Spielmann T. (2019). PfVPS45 is required for host cell cytosol uptake by malaria blood stage parasites. Cell Host Microbe.

[34] McGovern O.L., Rivera-Cuevas Y., Carruthers V.B. (2021). Emerging mechanisms of endocytosis in *Toxoplasma gondii*. Life.

[35] Quevillon E., Spielmann T., Brahimi K., Chattopadhyay D., Yeramian E., Langsley G. (2003). The *Plasmodium falciparum* family of Rab GTPases. Gene.

[36] Jackson A.J., Clucas C., Mamczur N.J., Ferguson D.J., Meissner M. (2013). *Toxoplasma gondii* Syntaxin 6 is required for vesicular transport between endosomal-like compartments and the Golgi complex. Traffic.

[37] Xie S.C., Dogovski C., Hanssen E., Chiu F., Yang T., Crespo M.P., Stafford C., Batinovic S., Teguh S., Charman S. (2016). Haemoglobin degradation underpins the sensitivity of early ring stage *Plasmodium falciparum* to artemisinins. J. Cell Sci..

[38] Birnbaum J., Scharf S., Schmidt S., Jonscher E., Hoeijmakers W., Flemming S., Toenhake C.G., Schmitt M., Sabitzki R., Bergmann B. (2020). A Kelch13-defined endocytosis pathway mediates artemisinin resistance in malaria parasites. Science.

[39] Ariey F., Witkowski B., Amaratunga C., Beghain J., Langlois A.C., Khim N., Kim S., Duru V., Bouchier C., Ma L. (2014). A molecular marker of artemisinin-resistant *Plasmodium falciparum* malaria. Nature.

[40] Rudzinska M.A. (1976). Ultrastructure of intraerythrocytic *Babesia microti* with emphasis on the feeding mechanism. J. Protozool..

[41] Rudzinska M.A., Trager W., Lewengrub S.J., Gubert E. (1976). An electron microscopic study of *Babesia microti* invading erythrocytes. Cell Tissue Res..

[42] Jalovecka M., Hajdusek O., Sojka D., Kopacek P., Malandrin L. (2018). The complexity of piroplasms life cycles. Front. Cell. Infect. Microbiol..

[43] Frerichs W.M., Holbrook A.A. (1974). Feeding mechanisms of *Babesia equi*. J. Protozool..

[44] Higuchi S., Kawamura S., Hanamatsu K., Yasuda Y. (1984). Electron microscopy of *Theileria sergenti* in bovine erythrocytes. Jpn. J. Vet. Sci..

[45] Fawcett D.W., Conrad P.A., Grootenhuis J.G., Morzaria S.P. (1987). Ultrastructure of the intra-erythrocytic stage of *Theileria* species from cattle and waterbuck. Tissue Cell.

[46] Langreth S.G. (1976). Feeding mechanisms in extracellular *Babesia microti* and *Plasmodium lophurae*. J. Protozool..

[47] Thekkiniath J., Kilian N., Lawres L., Gewirtz M.A., Graham M.M., Liu X., Ledizet M., Ben Mamoun C. (2019). Evidence for vesicle-mediated antigen export by the human pathogen *Babesia microti*. Life Sci. Alliance.

[48] Sun T., Tenenbaum M.J., Greenspan J., Teichberg S., Wang R.T., Degnan T., Kaplan M.H. (1983). Morphologic and clinical observations in human infection with *Babesia microti*. J. Infect. Dis..

[49] Taylor J.H., Guthrie A.J., Leisewitz A. (1991). The effect of endogenously produced carbon monoxide on the oxygen status of dogs infected with *Babesia canis*. J. S. Afr. Vet. Assoc..

[50] Goldberg D.E. (2005). Hemoglobin degradation. Curr. Top. Microbiol. Immunol..

[51] Saliba K.J., Allen R.J., Zissis S., Bray P.G., Ward S.A., Kirk K. (2003). Acidification of the malaria parasite’s digestive vacuole by a H+-ATPase and a H+-pyrophosphatase. J. Biol. Chem..

[52] Sullivan D.J., Gluzman I.Y., Goldberg D.E. (1996). *Plasmodium* hemozoin formation mediated by histidine-rich proteins. Science.

[53] Teixeira C., Gomes J.R., Gomes P. (2011). Falcipains, *Plasmodium falciparum* cysteine proteases as key drug targets against malaria. Curr. Med. Chem..

[54] Gluzman I.Y., Francis S.E., Oksman A., Smith C.E., Duffin K.L., Goldberg D.E. (1994). Order and specificity of the *Plasmodium falciparum* hemoglobin degradation pathway. J. Clin. Investig..

[55] Šnebergerová P., Bartošová-Sojková P., Jalovecká M., Sojka D. (2021). Plasmepsin-like aspartyl proteases in *Babesia*. Pathogens.

[56] Nasamu A.S., Polino A.J., Istvan E.S., Goldberg D.E. (2020). Malaria parasite plasmepsins: More than just plain old degradative pepsins. J. Biol. Chem..

[57] Dahl E.L., Rosenthal P.J. (2005). Biosynthesis, localization, and processing of falcipain cysteine proteases of *Plasmodium falciparum*. Mol. Biochem. Parasitol..

[58] Marco M., Coterón J.M. (2012). Falcipain inhibition as a promising antimalarial target. Curr. Top. Med. Chem..

[59] Becker K., Tilley L., Vennerstrom J.L., Roberts D., Rogerson S., Ginsburg H. (2004). Oxidative stress in malaria parasite-infected erythrocytes: Host-parasite interactions. Int. J. Parasitol..

[60] Chugh M., Sundararaman V., Kumar S., Reddy V.S., Siddiqui W.A., Stuart K.D., Malhotra P. (2013). Protein complex directs hemoglobin-to-hemozoin formation in *Plasmodium falciparum*. Proc. Natl. Acad. Sci. USA.

[61] De Villiers K.A., Egan T.J. (2021). Heme detoxification in the malaria parasite: A target for antimalarial drug development. Acc. Chem. Res..

[62] Fitch C.D., Cai G.Z., Chen Y.F., Shoemaker J.D. (1999). Involvement of lipids in ferriprotoporphyrin IX polymerization in malaria. Biochim. Biophys. Acta.

[63] Jani D., Nagarkatti R., Beatty W., Angel R., Slebodnick C., Andersen J., Kumar S., Rathore D. (2008). HDP—A novel heme detoxification protein from the malaria parasite. PLoS Pathog..

[64] Burda P.C., Crosskey T., Lauk K., Zurborg A., Söhnchen C., Liffner B., Wilcke L., Pietsch E., Strauss J., Jeffries C.M. (2020). Structure-based identification and functional characterization of a lipocalin in the malaria parasite *Plasmodium falciparum*. Cell Rep..

[65] Matz J.M., Drepper B., Blum T.B., van Genderen E., Burrell A., Martin P., Stach T., Collinson L.M., Abrahams J.P., Matuschewski K. (2020). A lipocalin mediates unidirectional heme biomineralization in malaria parasites. Proc. Natl. Acad. Sci. USA.

[66] Sigala P.A., Goldberg D.E. (2014). The peculiarities and paradoxes of *Plasmodium* heme metabolism. Annu. Rev. Microbiol..

[67] Ke H., Sigala P.A., Miura K., Morrisey J.M., Mather M.W., Crowley J.R., Henderson J.P., Goldberg D.E., Long C.A., Vaidya A.B. (2014). The heme biosynthesis pathway is essential for *Plasmodium falciparum* development in mosquito stage but not in blood stages. J. Biol. Chem..

[68] Nagaraj V.A., Sundaram B., Varadarajan N.M., Subramani P.A., Kalappa D.M., Ghosh S.K., Padmanaban G. (2013). Malaria parasite-synthesized heme is essential in the mosquito and liver stages and complements host heme in the blood stages of infection. PLoS Pathog..

[69] Goldberg D.E., Sigala P.A. (2017). *Plasmodium* heme biosynthesis: To be or not to be essential?. PLoS Pathog..

[70] Perner J., Gasser R.B., Oliveira P.L., Kopáček P. (2019). Haem Biology in Metazoan Parasites—‘The Bright Side of Haem’. Trends Parasitol..

[71] Cornillot E., Hadj-Kaddour K., Dassouli A., Noel B., Ranwez V., Vacherie B., Augagneur Y., Brès V., Duclos A., Randazzo S. (2012). Sequencing of the smallest apicomplexan genome from the human pathogen *Babesia microti*. Nucleic Acids Res..

[72] Florin-Christensen M., Wieser S.N., Suarez C.E., Schnittger L. (2021). In silico Survey and characterization of *Babesia microti* functional and non-functional proteases. Pathogens.

[73] Dhawan S., Dua M., Chishti A.H., Hanspal M. (2003). Ankyrin peptide blocks falcipain-2-mediated malaria parasite release from red blood cells. J. Biol. Chem..

[74] Mesplet M., Echaide I., Dominguez M., Mosqueda J.J., Suarez C.E., Schnittger L., Florin-Christensen M. (2010). Bovipain-2, the falcipain-2 ortholog, is expressed in intraerythrocytic stages of the tick-transmitted hemoparasite *Babesia bovis*. Parasites Vectors.

[75] Martins T.M., do Rosário V.E., Domingos A. (2011). Identification of papain-like cysteine proteases from the bovine piroplasm *Babesia bigemina* and evolutionary relationship of piroplasms C1 family of cysteine proteases. Exp. Parasitol..

[76] Martins T.M., do Rosário V.E., Domingos A. (2012). Expression and characterization of the *Babesia bigemina* cysteine protease BbiCPL1. Acta Trop..

[77] Okubo K., Yokoyama N., Govind Y., Alhassan A., Igarashi I. (2007). *Babesia bovis*: Effects of cysteine protease inhibitors on in vitro growth. Exp. Parasitol..

[78] Carletti T., Barreto C., Mesplet M., Mira A., Weir W., Shiels B., Oliva A.G., Schnittger L., Florin-Christensen M. (2016). Characterization of a papain-like cysteine protease essential for the survival of *Babesia ovis* merozoites. Ticks Tick-Borne Dis..

[79] Brayton K.A., Lau A.O., Herndon D.R., Hannick L., Kappmeyer L.S., Berens S.J., Bidwell S.L., Brown W.C., Crabtree J., Fadrosh D. (2007). Genome sequence of *Babesia bovis* and comparative analysis of apicomplexan hemoprotozoa. PLoS Pathog..

[80] Kloehn J., Harding C.R., Soldati-Favre D. (2021). Supply and demand-heme synthesis, salvage and utilization by Apicomplexa. FEBS J..

[81] Puri A., Bajpai S., Meredith S., Aravind L., Krause P.J., Kumar S. (2021). Pathogen Genomics, Genetic Variability, Immunodominant Antigens, and Pathogenesis. Front. Microbiol..

[82] Weiss L.M. (2002). Babesiosis in humans: A treatment review. Expert Opin. Pharmacother..

[83] Alves E., Maluf F.V., Bueno V.B., Guido R.V., Oliva G., Singh M., Scarpelli P., Costa F., Sartorello R., Catalani L.H. (2016). Biliverdin targets enolase and eukaryotic initiation factor 2 (eIF2α) to reduce the growth of intraerythrocytic development of the malaria parasite *Plasmodium falciparum*. Sci. Rep..

